# A rare case report of mucormycosis presenting as cranial nerve palsies in a patient with diabetes and iatrogenic cushing syndrome

**DOI:** 10.1097/MD.0000000000043886

**Published:** 2025-08-22

**Authors:** Farah Sadiq, Ali Gohar, Muhammad Husnain Ahmad, Masab Ali, Asad Ullah Khan, Bilal Ahmed, Fida Hussain, Muhammad Asfand Nadeem

**Affiliations:** a Department of Internal Medicine, Lahore General Hospital, Lahore, Punjab, Pakistan; b Department of Internal Medicine, S Tentishev Asian Medical Institute, Kant, Chuy Province, Kyrgyzstan; c Department of Internal Medicine, Punjab Medical College, Faisalabad, Punjab, Pakistan; d Department of Internal Medicine, Allied hospital, Faisalabad, Pakistan; e King Edward Medical University, Lahore, Punjab, Pakistan.

**Keywords:** abducens nerve palsy, diabetes mellitus, facial nerve palsy, immunocompromised, mucormycosis, ophthalmoplegia

## Abstract

**Rationale:**

Mucormycosis is a rare opportunistic fungal infection caused by mucormycetes, primarily affecting immunocompromised individuals such as diabetic patients. Cranial nerve involvement, including facial and abducens nerve palsies, is an uncommon but serious complication.

**Patient concerns:**

We present the case of a 47-year-old female with uncontrolled diabetes and iatrogenic Cushing’s syndrome who developed mucormycosis complicated by right-sided facial weakness (upper motor neuron type) and abducens nerve palsy (lower motor neuron type). She presented with a 1-month history of right-sided facial swelling, headache, and earache, and a 6-day history of sudden-onset facial weakness. Examination revealed preserved eye closure and forehead wrinkling, with a deficit in right eye abduction. Black eschar was noted in the intranasal and hard palate areas.

**Diagnoses:**

Imaging (computed tomography paranasal sinuses and magnetic resonance imaging) and histopathological confirmation established the diagnosis.

**Interventions:**

Treatment included amphotericin B and 2 surgical debridements following otorhinolaryngology consultation.

**Outcome:**

Patient’s facial swelling improved during hospital stay, and abduction deficit resolved on 1-month follow-up.

**Lessons:**

This case highlights the critical need for early recognition and multidisciplinary management of mucormycosis, particularly in diabetic or immunocompromised patients presenting with cranial nerve deficits. Prompt diagnosis and treatment are vital to reduce morbidity and improve outcomes.

## 1. Introduction

Mucormycosis is an uncommon yet serious fungal infection that primarily affects individuals with compromised immune systems. It is caused by fungi from the mucormycetes family, which typically colonize the nasal turbinates. Due to its proximity to vital structures like the orbit, sinuses, and brain, mucormycosis can present with a variety of clinical symptoms. A characteristic feature is the development of black eschar on affected tissues, which signifies extensive tissue necrosis.^[1]^ Based on the region of involvement, the infection is categorized into rhino-orbital-cerebral, pulmonary, cutaneous, gastrointestinal, and disseminated forms, each presenting distinct challenges. Severe cases may involve arterial, neural, and bony structures, leading to poor outcomes.^[2]^

Uncontrolled diabetes mellitus, especially with diabetic ketoacidosis, is the most significant risk factor for mucormycosis. Other contributing factors include hematological malignancies, prolonged use of corticosteroids, iron overload, and deferoxamine therapy, which facilitates fungal growth by acting as a siderophore.^[3]^ Studies have shown that mortality rates remain high, underscoring the importance of timely diagnosis and intervention.^[4]^ Diagnostic tools such as computed tomography (CT) paranasal sinuses (PNS) and magnetic resonance imaging are essential for assessing disease extent, while definitive diagnosis relies on lesion culture and biopsy. Effective treatment requires a combination of intravenous amphotericin B, repeated surgical debridement, and coordinated multidisciplinary care involving otorhinolaryngology specialists.^[5]^

Occasionally, mucormycosis may extend to nearby cranial nerves, leading to complications such as facial nerve palsy and ophthalmoplegia. The mechanisms underlying cranial nerve involvement are not fully understood but may involve direct fungal spread through the Eustachian tube, orbital cavity, or pterygopalatine fossa, as well as vascular invasion causing ischemic nerve damage.^[2,6]^ These complications increase morbidity and necessitate urgent medical attention.

Involvement of cranial nerve VI and VII was rarely reported in the literature.^[7]^ This case report describes a female patient with poorly controlled diabetes and cushingoid features, who developed intranasal and intraoral mucormycosis with complications involving the facial nerve (UMN type) and abducens nerve (LMN type). The report emphasizes the significance of early detection and comprehensive management in improving patient outcomes.

## 2. Case description

A 47-year-old female patient with uncontrolled diabetes presented to the medical outpatient department with right-sided facial swelling for 1 month. This was associated with headache and earache. She also complained of right-sided facial weakness for the last 6 days, which was sudden in onset, nonprogressive, and associated with drooling of saliva and difficulty in eating. There was no history of fever, trauma, ear discharge, or toothache. However, the patient had a history of steroid intake. She was taking oral glimepiride 2mg before breakfast for diabetes and prednisolone 5mg twice daily for last 2 years.

**Figure 1.**
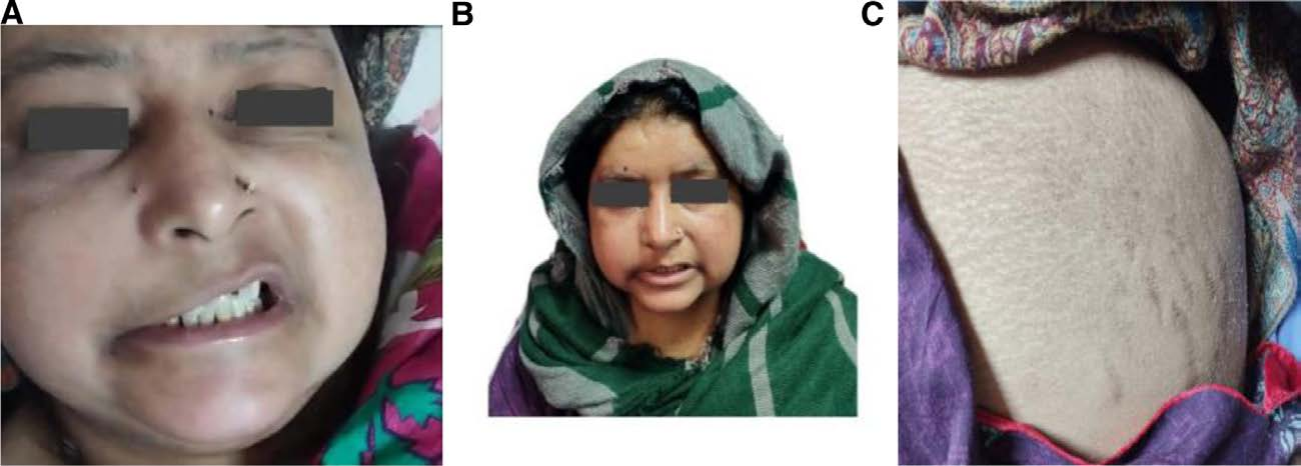
(A) Deviation of the angle of the mouth. (B) Moon faces and hirsutism. (C) Abdominal striae.

On examination, the patient’s glasgow coma scale was 15/15, and she was oriented to time, space, and person. Facial examination revealed drooping of the corner of the mouth on the right side, drooling of saliva, and an absence of wrinkles on the right half of the forehead, although eye closure was normal (Fig. 1A). There were no exophthalmos present. There was complete loss of abduction on extraocular movements of the right eye (suggestive of a lower motor neuron lesion). There was no pain but diplopia on lateral gaze. Cranial nerve examination revealed involvement of the VI and VII cranial nerves, but there was no alteration or loss of taste sensation. Intraoral and nasal examination revealed the presence of black eschar. Other clinical examination findings included moon face, hirsutism, truncal obesity, and abdominal striae (Fig. 1B and C).

**Figure 2.**
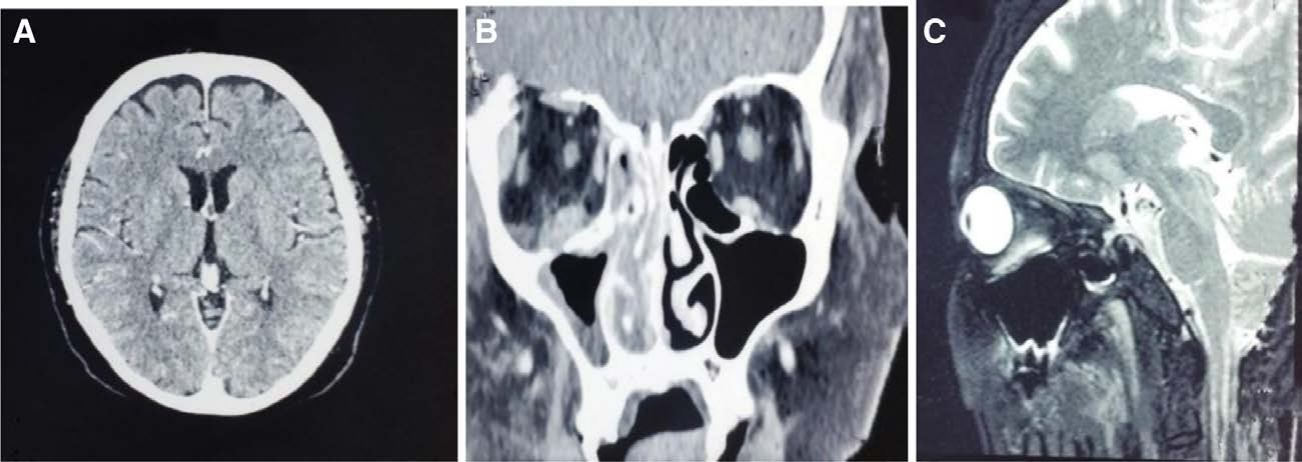
(A) Plain CT brain. (B and C) CT of PNS. CT = computed tomography, PNS = paranasal sinuses.

A differential diagnosis of orbital cellulitis and mucormycosis complicated with cranial nerve palsies and iatrogenic cushing was made. The departments of ENT, ophthalmology, and neurology were consulted. Routine biochemistry results are summarized in Table 1. Radiological investigations (Fig. 2A) revealed normal CTA and CTV findings. CT PNS (Fig. 2B) showed mild asymmetrical enlargement of the right lacrimal gland and bilateral maxillary, ethmoid, and right sphenoid sinusitis. magnetic resonance imaging brain with contrast demonstrated mucosal thickening of the right maxillary, sphenoid, ethmoidal, and frontal sinuses.

**Table 1:** Laboratory investigations.

Complete blood picture	Results	Reference ranges
HB	10.2 g/dL	12.0–15.5 g/dL
MCV	84.2 fL	80–100 fL
ESR	33 mm/h	**0–20 mm/h**
CRP	20 mg/L	< 10 mg/L
PLT	399 k/mcL	150–400 k/mcL
TLC	15.4 k/mcL	4.0–11.0 k/mcL
Coagulation profile		
PT	14 s	11–13.5 s
APTT	35 s	25–35 s
INR	1.0	**0.8–1.2**
Viral markers		
HbsAg	Negative	Negative
Anti HCV	Negative	Negative
Anti HIV	Negative	Negative
LFTs		
Total BIL	0.8 mg/Dl	0.1–1.2 mg/dL
ALT	26 IU/L	7–56 U/L
AST	36 IU/L	10–40 U/L
ALP	188 IU/L	44–147 U/L
Serum albumin	28 g/L	3.5–5.0 g/dL
Renal function tests		
Urea	34 mg/dL	15–40 mg/dL
Creatinine	0.7 mg/Dl	0.6–1.1 mg/dL
HbA1c	8.9	< 5.7%
D dimers	<200	< 500 ng/mL
IgE levels	27.44	0–100 IU/mL
Cardiac enzymes		
Serum MB	08 U/L	0–24 U/L
Serum LDH	273 U/L	140–280 U/L
Serum CK	41 U/L	26–140 U/L
Serum electrolytes		
Na+	136 mEq/L	135–145 mEq/L
K+	3.3 mEq/L	3.5–5.0 mEq/L
Ca++	2.27 mmol/L	2.12–2.62 mmol/L
Mg+	0.57 mmol/L	0.7–1.1 mmol/L
PO4	0.99 mmol/L	0.81–1.45 mmol/L
Urine complete examination		
Color	Yellow	Straw/pale yellow
Sp. gravity	1.020	1.005–1.030
PH	6.0	4.5–8.0
Protein/glucose	Nil	Negative
Pus cells	2–4	0–5/HPF
Blood	Nil	Negative
Red cells	01–02	0–2/HPF
Epithelial cells	04–06	0–5/HPF

ESR suggests chronic inflammation/infection, indicated in bold.

ALP = alkaline phosphatase, ALT = alanine aminotransferase, APTT = activated partial thromboplastin time, AST = aspartate aminotransferase, BIL = bilirubin, CK = creatine kinase, CRP = C-reactive protein, ESR = erythrocyte sedimentation rate, HB = hemoglobin, HbA1c = glycated hemoglobin, HbsAg = hepatitis B surface antigen, HCV = hepatitis C virus, HIV = human immunodeficiency virus, HPF = high power field, IgE = immunoglobulin E, INR = international normalized ratio, LDH = lactate dehydrogenase, LFTs = liver function tests, MB = myoglobin, MCV = mean corpuscular volume, PLT = platelet count, PT = prothrombin time, TLC = total leukocyte count.

**Figure 3.**
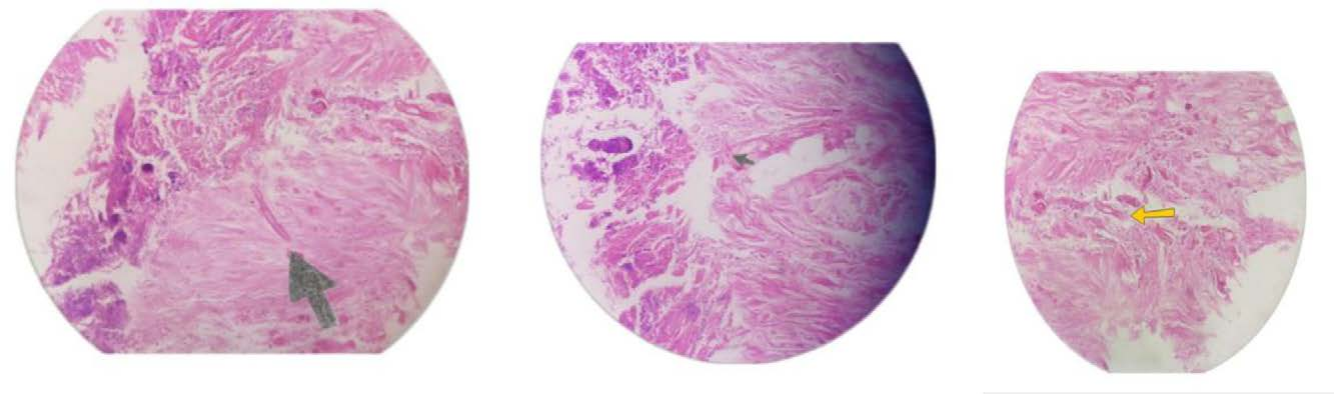
Fungal hyphae budding at wide angle against necrotic background (shown in yellow and green arrows).

A buccal biopsy was performed, and microscopic examination of the biopsy material and nasal discharge was conducted using 10% KOH wet mounts. The findings revealed necrotic patches of nonsporing, ribbon-like, broad, aseptate, branched hyphae, consistent with mucormycosis (Fig. 3). No granuloma or malignant cells were observed. The patient was managed medically with amphotericin B at 0.7 mg/kg/day, with close monitoring of serum electrolytes, blood sugar levels, and renal function. There was considerable improvement in facial swelling after twenty days’ stay at the hospital. After 1 month of follow-up, her symptoms were improved, and the abduction deficit of the right eye was completely resolved.

## 3. Discussion

Mucormycosis, a life-threatening fungal infection caused by fungi of the order *Mucorales*, predominantly affects immunocompromised individuals. In the presented case, the 47-year-old diabetic patient with poorly controlled glycemia developed rhino-orbital-cerebral mucormycosis, an aggressive manifestation characterized by sinusitis, cranial nerve palsies, and black eschar. Uncontrolled diabetes, particularly in ketoacidosis, impairs neutrophilic function and increases fungal proliferation in hyperglycemic, acidic environments. Mane et al^[1]^ highlighted the vulnerability of diabetic patients to rhino-orbital mucormycosis, while clinical features such as moon face and truncal obesity suggest concomitant Cushing syndrome, exacerbating immunosuppression.

Cranial nerve involvement, including VI and VII palsies, reflects the invasive nature of mucormycosis. Barahimi et al^[5]^ noted fungal infiltration into the orbital apex or cavernous sinus as a cause of nerve dysfunction. This aligns with the patient’s facial weakness and absence of forehead wrinkles, consistent with lower motor neuron facial nerve palsy. Mehdi et al^[3]^ described similar presentations with facial palsy and total ophthalmoplegia, emphasizing the importance of recognizing atypical presentations.

Spellberg et al^[2]^ described mucormycosis as angioinvasive, leading to thrombosis and necrosis of infected tissues, explaining the black eschar seen in this case. This invasive nature necessitates early diagnosis and treatment. Roden et al^[4]^ reviewed over 900 cases, demonstrating that delayed intervention significantly worsens outcomes. Rapid diagnosis in this patient was achieved through imaging and KOH microscopy, which demonstrated broad, aseptate hyphae consistent with mucormycosis.

Management included liposomal amphotericin B, administered at 0.7 mg/kg/day, the first-line antifungal agent. Mane et al^[1]^ and Mehdi et al^[3]^ emphasized its role in halting fungal progression. Glycemic control and electrolyte monitoring were crucial to mitigating adverse effects. Surgical debridement, a cornerstone of management in many cases,^[2]^ was not required here due to the patient’s prompt response to medical therapy. The patient’s clinical improvement highlights the efficacy of early and aggressive treatment.

Mucormycosis carries high morbidity and mortality, particularly with extensive cranial nerve involvement. Regular follow-up is critical to monitor for recurrence or residual deficits. Barahimi et al stressed long-term surveillance in immunocompromised patients to ensure sustained recovery and prevent complications.^[5]^

This case points towards the need for educational awareness among diabetic and immunocompromised patients on the initial clinical manifestations of mucormycosis, including facial pain, numbness, or swelling, nasal congestion or dripping, vision problems, and headaches. They should also be well-aware of the significance of early medical intervention if they come across any of these symptoms, especially if they have a history of poorly controlled diabetes or decreased immunity. Patients should be trained and educated to get immediate medical help if they notice any unusual problem, as early diagnosis and treatment are critical in managing mucormycosis effectively. Regular monitoring of blood sugar levels, good hygiene practices, and adherence to medication regimens can also help reduce the risk of developing mucormycosis.

## 4. Conclusions

In conclusion, this case underscores the pivotal role of rapid detection, timely antifungal therapy, and strict metabolic control in managing mucormycosis effectively. Multidisciplinary collaboration enhances patient outcomes by addressing complex presentations involving multiple systems. Regular follow-up is essential for monitoring residual disease and ensuring sustained recovery. Greater awareness and continued research into novel diagnostic and therapeutic approaches are imperative to combat the high mortality and morbidity associated with this invasive fungal infection. Education and training for healthcare professionals are also vital in improving the early recognition and management of such cases.

## Acknowledgments

We would like to thank the team of clinicians who helped manage this case. We would like to thank the patient and his family members for their cooperation in bringing this case for the betterment of the scientific community.

## Author contributions

**Conceptualization:** Farah Sadiq.

**Validation:** Ali Gohar, Muhammad Husnain Ahmad, Masab Ali, Fida Hussain, Muhammad Asfand Nadeem.

**Visualization:** Masab Ali, Asad Ullah Khan, Bilal Ahmed, Fida Hussain, Muhammad Asfand Nadeem.

**Writing – original draft:** Farah Sadiq, Masab Ali, Asad Ullah Khan.
